# Mental health disorders, childhood adversities, and recent stressors as risk factors for non-suicidal self-injury, and suicidality among LGBTQA + higher education students

**DOI:** 10.1186/s12889-025-23102-7

**Published:** 2025-08-07

**Authors:** Emma Rebecca Wallace, Margaret McLafferty, Rachel McHugh, Caoimhe Ward, Louise McBride, John Brady, Susan Lagdon, Anthony J. Bjourson, Colum P. Walsh, Siobhan O’Neill, Elaine K. Murray

**Affiliations:** 1 School of Psychology, Ulster University, Cromore road, Coleraine, Northern Ireland UK; 2 Personalised Medicine Centre, School of Medicine, Ulster University, Derry-Londonderry, Ireland; 3 Department of Nursing and Health Care, Atlantic Technological University Donegal, Letterkenny, Ireland; 4 Adult Psychiatry, Western Health and Social Care Trust, Omagh, Northern Ireland UK; 5 Department of Biomedical and Clinical Sciences, Linköping University, Linköping, SE-581 83 Sweden

**Keywords:** Higher education students, LGBTQA+, Northern Ireland, Republic of Ireland, Mental health disorders, Non-suicidal self-injury, Suicidality, Risk factors, Childhood adversities, Recent stressors.

## Abstract

**Background:**

Prevalence rates of mental health disorders, non-suicidal self-injury and suicidality among Lesbian, Gay, Bisexual, Transgender, Queer and Asexual (LGBTQA+) higher education students are consistently higher than rates for heterosexual students. Nevertheless, in the United Kingdom, there remains limited prevalence data and evidence on the risk factors that confer increased risk of suicide among this population. The purpose of the present study was to investigate mental health disorders, childhood adversities, and recent stressors as risk factors for non-suicidal self-injury, and suicidality among LGBTQA + students.

**Methods:**

The Student Psychological Interventional Trial (SPIT) was conducted as part of the World Mental Health International College Student Initiative (WMH-ICS). First year undergraduate students aged 18–24 years were recruited (*n* = 1525), including LGBTQA + students (*n* = 190). Chi-squared test of independence was used to identify significant differences in lifetime prevalence rates between heterosexual and LGBTQA + students. Bivariate and multivariate logistic regression analyses examined the associations between mental health disorders, childhood adversities, recent stressors, non-suicidal self-injury, and suicidality among LGBTQA + students.

**Results:**

LGBTQA + students were significantly more likely to have experienced mental health difficulties, childhood adversities, recent stressors, non-suicidal self-injury, and suicidality than their heterosexual counterparts. One in four LGBTQA + students reported experiencing major depressive episode, and non-suicidal self-injury in their lifetime. LGBTQA + students also reported earlier onset of mental health disorders and suicidality, with much higher rates of interpersonal conflict, and probable post-traumatic stress disorder. Among LGBTQA + students, major depressive disorder, probable post-traumatic stress disorder, and non-suicidal self-injury were significantly associated with an increased likelihood of suicidal ideation and suicide plan. Childhood adversities, and recent stressors such as bullying were significantly associated with an increased likelihood of suicide attempt.

**Conclusions:**

Our findings highlight the existing mental health disparities, childhood adversities, and recent stressors which may contribute to higher prevalence rates of non-suicidal self-injury, and suicidality among LGBTQA + students. The results emphasise the importance of early intervention, prevention, and treatment, focused on reducing the impact of childhood adversities and recent stressors such as bullying. In addressing these risk factors, educational settings may offer unique opportunities for the practice of inclusion, preventative care, and harm reduction for LGBTQA + students.

## Background

Every single life lost to suicide is a tragedy; suicide is preventable and not inevitable. Yet globally, suicide is the fourth leading cause of death among young people aged 15–29 years old [1]. Northern Ireland (NI) and the Republic of Ireland (ROI) continue to be impacted by high suicide rates, with an average of 14.3 deaths per 100,000 in 2021 [2], and the standardised average of 9.2 deaths per 100,000 in 2021 respectively [3]. Several studies worldwide have reported on higher prevalence rates of suicidality among marginalised groups, including LGBTQ + young people [4–6]. For example, a recent systematic review found that lesbian, gay and bisexual youth were three times more likely than their heterosexual peers to have made a suicide attempt in their lifetime [7]. Although one of the most prominent predictors of suicidality is a previous history of suicidal behaviour [8], it is widely accepted that suicide is a complex, multifactorial and highly contextual phenomenon [9]. Nevertheless, the existence of higher prevalence rates of mental health disorders, including depression and anxiety, among LGBTQ + youth is a significant risk factor for non-suicidal self-injury (NSSI) and suicidality [10, 11]. Moreover, existing literature has emphasised the pervasive impact of childhood adversities, which are distinctly higher within the LGBTQ + community [12]. It follows then that higher rates of interpersonal trauma and victimisation may result in poorer mental health outcomes, including suicidality among LGBTQ + youth [13, 14].

Crucially, for young adults beginning higher education these mental health difficulties may precede, and may indeed worsen with, the challenging transition into adulthood and independent student life. Indeed, academic pressures and having to adjust to a new educational experience can raise prevalence rates of stress, depression and anxiety, particularly for students from disadvantaged backgrounds [15, 16]. In fact, the Ulster University Student Wellbeing Study (UUSWS) reported that baseline prevalence rates of both lifetime and 12-month mental health disorders and suicidal behaviour were high among higher education students [17]. In the ROI, a cross-sectional study among undergraduate students also found that 59% of students had experienced depressive symptoms that may require treatment, and 28.5% reported suicidal ideation [18, 19]. Additionally, a recent systematic review on higher education students found that students most at risk of developing mental health difficulties were LGBTQ + students, and those who had experienced significant childhood adversities [20]. Importantly, it has also been found that LGBTQ + higher education students are three times more likely to have experienced mental health disorders and suicidality than their heterosexual peers [17, 19, 21]. Critically though, previous studies have not examined the complex factors which may confer increased risk of suicidality among LGBTQA + higher education students.

These findings demonstrate the poor mental health of higher education students, exemplifying the importance of understanding risk factors associated with both NSSI and suicidality. However, there remains limited local evidence on the mental health disparities and increased risk of suicidality among Lesbian, Gay, Bisexual, Transgender, Queer, and Asexual (LGBTQA+) higher education students. In order to address this empirical gap, the present study investigated prevalence rates of mental health disorders, childhood adversities and recent stressors as risk factors for NSSI and suicidality among LGBTQA + students, compared to their heterosexual peers.

## Method

### Design

The Student Psychological Interventional Trial (SPIT) was a two-phase project funded by the Cross-border Healthcare Intervention Trials in Ireland Network (CHITIN) funded project. The first phase commenced in September 2019 with its recruitment of higher education students across all four campuses of Ulster University (Derry/Londonderry, Coleraine, Jordanstown and Belfast), and Letterkenny Institute of Technology (LYIT) in County Donegal, Republic of Ireland. This study was conducted as part of the World Mental Health International College Student Initiative (WMH-ICS) [22].

### Sample

One week prior to registration, prospective students were emailed the participant information sheet which detailed the study aims and methodology. Trained volunteers recruited students across UU and LYIT campuses following the registration process. According to WMH-ICS protocols, only students who were 18 years old or over and who resided in Northern Ireland (NI), or the Republic of Ireland (ROI) were eligible to participate. As such, international students, postgraduate students, or those repeating the first year of their undergraduate studies were excluded. Students who provided written informed consent were given an online survey link and a unique participant code in order to take part, and those who fully completed the survey received a branded university or college sweatshirt. The mental health survey was administered online using Qualtrics software. In total, 1828 students fully completed the online mental health survey, including 1468 from NI and 360 from the ROI.

For the current analysis, participants were 1525 students with similar sample demographics for both heterosexual (*n* = 1335) and LGBTQA + students (*n* = 190). The majority of students who participated in this study were from Ulster University (*n* = 1208). Among heterosexual students, there were female students (*n* = 742) and male students (*n* = 593). Among LGBTQA + students, there were female (cisgender) students (*n* = 113), male (cisgender) students (*n* = 74), transgender males (*n* = 2), and one student who identified as gender-diverse (*n* = 1). Among heterosexual students, 83.1% were aged under twenty-one years, with 85.8% among LGBTQA + students. The vast majority of students were unmarried (*n* = 1323), representing 99.5% of both heterosexual and LGBTQA + higher education students. The completed response rate for UU was 25.22% of the total intake, and 41.9% for LYIT among students from courses who were invited to participate.

### Survey

The validated survey was adapted from the WMH-Composite International Diagnostic Interview (WMH-CIDI) [23], and developed by the WMH-ICS. The survey was used to explore the prevalence of mental health disorders in accordance with DSM-V criteria. The instrument includes screening for major depressive disorder, major depressive episode, probable post-traumatic stress disorder (PTSD), anxiety disorders, and emotional wellbeing. Suicidal thoughts, plans and attempts, both lifetime and within the past 12 months, were assessed using items from the Self-Injurious Thoughts and Behaviour Interview (SITBI) [24]. The instrument has good psychometric properties in relation to both reliability and validity [24]. Information about support resources were provided to all participants, and the university counselling services were alerted if a participant indicated that they had either planned or attempted suicide in the past year. In these cases, students were contacted and assessed by trained counsellors who provided support where required. NSSI was also assessed using items from the Self-Injurious Thoughts and Behaviour Interview (SITBI) [24].

Probable post-traumatic stress disorder (PTSD) was a screening item which was assessed according to self-reported symptoms, lasting for more than one month, following an extremely stressful experience. Symptoms included frequent upsetting memories or dreams, feeling jumpy, emotionally distant or depressed, and significant trouble sleeping or concentrating. In the current study, ten childhood adversities including both parental maltreatment and parental maladjustment were measured. Parental maltreatment included physical abuse, emotional abuse, neglect, emotional closeness and feeling loved or cared for at home. Parental maladjustment included parental mental illness, parental suicide attempt, parental substance misuse, and domestic violence. Parental mental health problems and substance misuse were measured using questions developed for the Army STARRS project [25]. Parental suicide attempt and childhood neglect were measured using an adapted version of the childhood section of the WMH-CIDI [26]. Domestic violence, physical abuse, and emotional abuse were measured using questions adapted from the Adverse Childhood Experiences Scale [27]. Self-reported recent stressors were also investigated, including a number of stressful experiences such as interpersonal conflict, family relationship stress, overall health stress, sexual harassment, and bullying.

### Data analysis

Weights were applied in all data analyses to ensure that the results were representative of the total student population. These weights were created using the gender and age characteristics of the first-year student population in both NI and the ROI. First, Chi-square test of independence was used to identify significant lifetime differences between heterosexual and LGBTQA + students, including mental health disorders, probable PTSD, NSSI, and suicidality (ideation/plan/attempt). Similarly, childhood adversities and recent stressful experiences were also investigated. Second, the age of onset, defined by the first time a participant reported experiencing difficulties, mental health disorders, NSSI, and suicidality were examined with independent samples *t*-tests to determine differences. Third, bivariate and multi-variate odds ratio logistic regression analyses were used to explore associations between NSSI, suicidality and a range of identified independent variables. This included mental health disorders, probable PTSD, childhood adversities, and recent stressors. All statistical analyses were carried out using SPSS (version 29). Listwise deletion was used to handle any missing data, excluding cases (*n* = 22) from the overall sample with any missing values for the primary outcome variables used in the analysis. Furthermore, post-hoc analyses involving a False Discovery Rate (FDR) correction were performed, employing the Benjamini-Hochberg method, with the widely accepted and appropriate q-value of 0.05 [28, 29].

## Results

### Prevalence of lifetime mental health disorders, non-suicidal self-injury and suicidality

Overall, the highest self-reported lifetime prevalence rates examining heterosexual and LGBTQA + students together (Table 1) were for probable PTSD (39.1%), suicidal ideation (27.7%), major depressive episode (14.4%), and non-suicidal self-injury (14.4%). In a comparison of lifetime prevalence rates between heterosexual and LGBTQA + students, LGBTQA + students had experienced significantly higher rates of probable PTSD, non-suicidal self-injury (NSSI), major depressive episode (MDE), and suicidality (ideation/plan/attempt). The lifetime prevalence rates of probable PTSD among LGBTQA + students was 51.1%, compared to 37.4% among heterosexual students, χ2 (1, *n* = 1525) = 13.063, *p* = < 0.001, phi = 0.281. The lifetime prevalence rates of NSSI among LGBTQA + students was 28.4%, compared to 12.4% among heterosexual students, χ2 (1, *n* = 1525) = 34.952, *p* = < 0.001, phi = 0.258. The lifetime prevalence rates of MDE among LGBTQA + students was 26.3%, compared to 12.6% of heterosexual students, χ2 (1, *n* = 1525) = 25.275, *p* = < 0.001, phi = 0.261.

The lifetime prevalence of suicidal ideation among LGBTQA + students was 46.6%, compared to 25.1% of heterosexual students, χ2 (1, *n* = 1525) = 38.135, *p* = < 0.001, phi = 0.183. The lifetime prevalence of suicide plan among LGBTQA + students was 30.5%, compared to 11.7% of heterosexual students, χ2 (1, *n* = 1525) = 49.020, *p* = < 0.001, phi = 0.212. The lifetime prevalence of suicide attempt among LGBTQA + students was 14.2%, compared to 5.5% of heterosexual students, χ2 (1, *n* = 1525) = 20.238, *p* = < 0.001, phi = 0.174. The observed effect sizes, using the phi coefficient, indicate a small to medium effect size for the prevalence of mental health disorders, non-suicidal self-injury, and suicidality among higher education students. These results are presented in Table 1 below.

**Table 1:** Comparison of lifetime prevalence of mental health disorders, non-suicidal self-injury, suicide ideation, plan, and attempt among heterosexual and LGBTQA + students

Disorders	Total		Hetero		LGBTQA+		
	Total	(1525)		(1335)		(190)	
	n	%	n	%	n	%	*χ2*
MDE	219	14.4	169	12.6	50	26.3	25.275***
Probable PTSD	596	39.1	499	37.4	97	51.1	13.063***
NSSI	219	14.4	165	12.4	54	28.4	34.952***
Suicide ideation	423	27.7	335	25.1	88	46.6	38.135***
Suicide plan	214	14	156	11.7	58	30.5	49.020***
Suicide attempt	101	6.6	74	5.5	27	14.2	20.238***

*Note: n =* raw unweighted values, % weighted values. MDE = major depressive episode, PTSD = probable post-traumatic stress disorder, NSSI = non-suicidal self-injury. *χ2* shows significant differences in prevalence rates. ***p < 0.001

### Age of onset for mental health disorders, non-suicidal self-injury and suicidality

The average age of onset for self-reported mental health disorders, NSSI and suicidality overall in the student population was between the ages of twelve and fifteen (Table 2). An independent samples t-test was conducted to compare the age of onset for major depressive disorder (MDE) between LGBTQA + students (M = 12.81, SD = 3.067) and heterosexual students (M = 14.39, SD = 3.260). The results revealed a significant difference between the groups, *t* = 3.024, *p* = 0.003. The effect size, as measured by Cohen’s d, was d = 0.48 indicating a small to medium effect. A further analysis was conducted to compare the age of onset for suicidal ideation between LGBTQA + students (M = 13.73, SD = 3.811) and heterosexual students (M = 15.03, SD = 2.841). The results revealed a significant difference between the groups, *t* = 3.509, *p* = < 0.001. The effect size, as measured by Cohen’s d, was d = 0.42 indicating a small to medium effect. There were no significant differences reported between LGBTQA + and heterosexual students in the age of onset for NSSI, suicide plan, or suicide attempt. These results are presented in Table 2.

**Table 2:** Comparison of age of onset for mental health disorders, non-suicidal self-injury, and suicidality among heterosexual and LGBTQA + student

Disorders	Heterosexual	SD	LGBTQA+	SD
	Total Mean		Total Mean	
	Age		Age	
MDE	14.39	3.260	12.81	3.067**
NSSI	14.91	2.415	13.66	3.169
Suicide ideation	15.03	2.841	13.73	3.811***
Suicide plan	15.27	2.774	14.41	3.562
Suicide attempt	15.57	2.799	15.58	2.395

Note: SD = standard deviation, MDE = major depressive episode, NSSI = non-suicidal self-injury. * *p* < 0.05, **p < 0.01, ***p < 0.001

### Prevalence of childhood adversities and recent stressors

The highest self-reported prevalence rates for childhood adversities among heterosexual and LGBTQA + students were for parental mental illness (6.4%), parental alcohol and/or drugs misuse (4.1%), emotional abuse (3.8%), and physical abuse (2.2%) (Table 3). In a comparison of prevalence rates between heterosexual and LGBTQA + students, LGBTQA + students had experienced significantly higher rates of parental maltreatment and maladjustment. The lifetime prevalence rates of physical abuse among LGBTQA + students was 6.3%, compared to 1.6% among heterosexual students, χ2 (1, *n* = 1525) = 45.965, *p* = < 0.001, phi = 0.270. The lifetime prevalence rates of emotional abuse among LGBTQA + students was 11.7%, compared to 2.6% among heterosexual students, χ2 (1, *n* = 1525) = 55.683, *p* = < 0.001, phi = 0.301. The lifetime prevalence rates of parental mental illness among LGBTQA + students was 11.1%, compared to 5.7% among heterosexual students, χ2 (1, *n* = 1525) = 24.721, *p* = < 0.001, phi = 0.301. The lifetime prevalence rates of parental substance misuse among LGBTQA + students was 7.9%, compared to 3.6% among heterosexual students, χ2 (1, *n* = 1525) = 26.862, *p* = < 0.001, phi = 0.278. The lifetime prevalence rates of domestic violence among LGBTQA + students was 5.6%, compared to 0.8% among heterosexual students, χ2 (1, *n* = 1525) = 28.876, *p* = < 0.001, phi = 0.305.

Overall, the highest self-reported prevalence rates of recent stressors among heterosexual and LGBTQA + students were for friends and/or family conflict (14.4%), bullying (11.6%), and sexual harassment (7.3%). In a comparison of prevalence rates between heterosexual and LGBTQA + students for recent stressors, LGBTQA + students had experienced significantly higher rates of interpersonal conflict, bullying, sexual harassment, and sexual assault. The lifetime prevalence rates for bullying among LGBTQA + students was 20.8%, compared to 10.3% among heterosexual students, χ2 (1, *n* = 1525) = 16.999, *p* = < 0.001, phi = 0.198. The lifetime prevalence rates for interpersonal conflict among LGBTQA + students was 19.3%, compared to 13.7% among heterosexual students, χ2 (1, *n* = 1525) = 4.039, *p* = < 0.05, phi = 0.289. The lifetime prevalence rates for sexual harassment among LGBTQA + students was 17.2%, compared to 5.9% among heterosexual students, χ2 (1, *n* = 1525) = 30.536, *p* = < 0.001, phi = 0.242. The lifetime prevalence rates for sexual assault among LGBTQA + students was 4.3%, compared to 1.9% among heterosexual students, χ2 (1, *n* = 1525) = 4.505, *p* = < 0.05, phi = 0.178.

In addition, the findings highlighted that LGBTQA + students felt less emotional closeness with their family members and felt less loved or cared for at home. The lifetime prevalence rates for emotional closeness among LGBTQA + students was 37.4%, compared to 50.5% among heterosexual students, χ2 (1, *n* = 1525) = 20.477, *p* = < 0.001, phi = 0.396. The lifetime prevalence rates for feeling loved or cared for at home among LGBTQA + students was 45.5%, compared to 54.3% among heterosexual students, χ2 (1, *n* = 1525) = 15.532, *p* = < 0.05, phi = 0.361. No significant differences were found across the LGBTQA + and heterosexual student samples in the prevalence rates of physical assault, and overall health stress. The observed effect sizes, using the phi coefficient, indicate a small to medium effect size for the prevalence of childhood adversities and recent stressors among higher education students. These results are presented in Table 3 below.

**Table 3:** Comparison of prevalence of childhood adversities and recent stressors among heterosexual and LGBTQA + students

	Total		Hetero		LGBTQA+		
	Total	(1525)		(1335)		(190)	
	n	%	n	%	n	%	*χ2*
*Childhood adversities*							
Physical abuse	33	2.2	21	1.6	12	6.3	45.965***
Emotional abuse	57	3.8	35	2.6	22	11.7	55.683***
Parental mental illness	97	6.4	76	5.7	21	11.1	24.721***
Parental alcohol/drugs	63	4.1	48	3.6	15	7.9	26.862***
Domestic violence	20	1.3	10	0.8	10	5.6	28.876***
Emotional closeness	740	48.8	670	50.5	70	37.4	20.477***
Felt loved/cared for	808	53.2	722	54.3	86	45.5	15.532*
*Recent stressors*							
Family/friend conflict	219	14.4	183	13.7	36	19.3	4.039*
Bullying	174	11.6	136	10.3	38	20.8	16.999***
Physical assault	37	2.5	29	2.2	8	4.3	3.055
Sexual assault	33	2.2	25	1.9	8	4.3	4.505*
Sexual harassment	110	7.3	78	5.9	32	17.2	30.536***
Health stress	53	3.5	40	3.0	13	6.9	14.967

*Note: n =* raw unweighted values, % weighted values. *χ2* shows significant differences in prevalence rates. *p < 0.05, **p < 0.01, ***p < 0.001

### Bivariate logistic regression analysis

Table 4 below illustrates the binary logistic regression analyses of lifetime mental health disorders, childhood adversities, recent stressors, NSSI, and suicidality among LGBTQA + students. It is important to note that bivariate analyses report on the initial associations between predictor variables and outcome measures, providing evidence for which variables may be appropriate for inclusion in multivariate models. These bivariate analyses are therefore most appropriately conceptualised as a precursor to the primary multivariate analyses. Experiences of major depressive episode were associated with an increased likelihood of suicide ideation, plan and attempt as-well as NSSI. Probable PTSD was also associated with increased likelihood of suicide ideation, plan, attempt and non-suicidal self-injury. NSSI significantly increased the likelihood of suicide ideation, plan and attempt among students. For childhood adversities and parental maltreatment, experiences of emotional abuse were associated with increased likelihood of suicide ideation, plan, attempt and NSSI. For parental maladjustment, experiences of parental mental illness were associated with increased likelihood of suicide ideation, plan, attempt and NSSI. Experiences of domestic violence were associated with an increased likelihood of suicide plan and suicide attempt. In relation to recent stressors, overall health stress was associated with increased likelihood of suicide ideation, plan, attempt and NSSI. Furthermore, experiences of being bullied was associated with increased the likelihood of suicide ideation, suicide plan, and suicide attempt among LGBTQA + students.

**Table 4:** Bivariate logistic regression analyses of lifetime mental health disorders, childhood adversities, recent stressors, non-suicidal self-injury and suicidality among LGBTQA + students

Demographics *N* = 190	Suicide ideation OR (95% CI)	Suicide plan OR (95% CI)	Suicide attempt OR (95% CI)	Non-suicidal self-injury OR (95% CI)
*Mental health disorders*				
Major depressive episode	7.244*** (3.346–15.683)	5.442*** (2.709–10.933)	4.785*** (2.040-11.226)	5.871*** (2.896–11.902)
Probable PTSD	5.839*** (3.113–10.952)	7.818*** (3.655–16.720)	7.423*** (2.384–23.119)	17.665*** (6.618–47.153)
Non-suicidal self-injury	9.286*** (4.253–20.276)	10.396*** (5.019–21.536)	17.168*** (6.076–48.511)	-
*Childhood adversities*				
Physical abuse	1.251 (0.890-1.759)	1.561* (1.101–2.213)	1.678 (1.159–2.265)	1.401 (0.996-1.969)
Emotional abuse	1.462* (1.095–1.953)	1.605** (1.206–2.137)	1.274** (1.274–2.432)	1.825** (1.356–2.456)
Parent with mental illness	1.352* (1.105–1.653)	1.501*** (1.215–1.854)	1.847*** (1.389–2.456)	1.563*** (1.258–1.940)
Parental criminal activity	1.270 (0.909-1.898)	1.588 (1.129–2.232)	1.713* (1.200-2.446)	1.585 (1.130–2.223)
Parental domestic violence	1.372 (0.992-1.898)	1.378* (1.012–1.877)	1.448* (1.025–2.046)	1.199 (0.876-1.639)
Recent stressors				
Health stress	2.042*** (1.480–2.817)	2.319*** (1.648–3.261)	2.393*** (1.575–3.635)	2.016*** (1.448–2.806)
Bullied stress	2.336* (1.121–4.870)	2.529* (1.214–5.268)	3.571* (1.480–8.616)	1.407 (0.657-3.014)

*Note.* OR = odds ratio, CI = confidence intervals, significance values. *p < 0.05, **p < 0.01, ***p < 0.001

### Multivariate logistic regression analyses

Table 5 below illustrates the multivariate logistic regression analyses of mental health disorders, probable PTSD, NSSI, and suicidality among LGBTQA + students. Experiences of major depressive disorder was associated with a significantly increased likelihood of suicidal ideation, and suicide plan. Experiences of probable PTSD was associated with a significantly increased likelihood of suicidal ideation, suicide plan, and NSSI. Experiences of NSSI was associated with a significantly increased likelihood of suicidal ideation, suicide plan, and suicide attempt among LGBTQA + students.

**Table 5:** Multivariate logistic regression analyses of mental health disorders, probable PTSD, non-suicidal self-injury and suicidality among LGBTQA + students

Predictor variables *N* = 190	Suicide ideation OR (95% CI)	Suicide plan OR (95% CI)	Suicide attempt OR (95% CI)	Non-suicidal self-injury OR (95% CI)
Major depressive disorder	4.695* (1.741–12.660)	3.018* (1.232–7.392)	1.004 (0.346-2.913)	2.148 (0.900-5.125)
Probable PTSD	2.869* (1.387–5.933)	3.449* (1.453–8.191)	2.061 (0.533-7.967)	15.968*** (5.942–42.912)
Non-suicidal self-injury	4.910*** (2.051–11.756)	5.410*** (2.395–12.222)	12.109*** (3.689–39.753)	-

*Note.* OR = odds ratio, CI = confidence intervals, significance values. *p < 0.05, **p < 0.01, ***p < 0.001

Table 6 below illustrates the multivariate logistic regression analyses of childhood adversities, recent stressors, NSSI, and suicidality among LGBTQA + students. For students who had experienced childhood adversities, experiences of parental maltreatment and maladjustment was associated with an increased likelihood of suicide attempt. For recent stressors, experiences of overall health stress was associated with an increased likelihood of suicidal ideation, suicide plan, suicide attempt, and NSSI. Moreover, the experience of being bullied was associated with a significantly increased likelihood of suicide attempt among LGBTQA + higher education students.

**Table 6:** Multivariate logistic regression analyses of childhood adversities, recent stressors, non-suicidal self-injury and suicidality among LGBTQA + students

Predictor variables *N* = 190	Suicide ideation OR (95% CI)	Suicide plan OR (95% CI)	Suicide attempt OR (95% CI)	Non-suicidal self-injury OR (95% CI)
Childhood adversities	1.304 (0.789-2.153)	1.592 (0.949-2.672)	2.685*** (1.488–4.846)	1.634 (0.984-2.712)
Health stress	1.847*** (1.283–2.659)	1.919*** (1.319–2.791)	2.128* (1.332-3.400)	1.692* (1.175–2.436)
Bullied stress	1.180	1.626	3.043***	0.797
	(0.661-3.434)	(0.700-3.775)	(1.077–8.599)	(0.331-1.919)

*Note.* OR = odds ratio, CI = confidence intervals, significance values. *p < 0.05, **p < 0.01, ***p < 0.001

## Discussion

The present study examined lifetime prevalence rates of mental health disorders, childhood adversities, and recent stressors as risk factors for non-suicidal self-injury (NSSI), and suicidality among LGBTQA + higher education students. The findings demonstrated that overall rates of mental health disorders, childhood adversities and recent stressors were high among all higher education students. However, lifetime prevalence rates of mental health disorders, non-suicidal self-injury, and suicidality were up to three times higher among LGBTQA + students, compared to heterosexual students, in accordance with extensive international evidence [4, 30, 31]. The finding that more than half of all LGBTQA + students (51.1%) had experienced probable post-traumatic stress disorder is significant, emphasising the association between post-traumatic stress disorder, interpersonal trauma, victimisation and increased risk for suicidality among LGBTQA + individuals [13, 32, 33]. Almost half of all LGBTQA + students had thought about suicide in their lifetime (46%), compared to just over a quarter of heterosexual students (27.7%), and almost a third had made a suicide plan (30%), compared to their heterosexual counterparts (11.7%). In addition, 14.2% of LGBTQA + students had made at least one suicide attempt in their lifetime, compared to heterosexual students (5.5%). These concerning findings are supported by limited local evidence, underscoring that LGBTQA + higher education students are disproportionally affected by suicidal thoughts and behaviours [19, 21]. Nevertheless, it is important to acknowledge that LGBTQA + students are not inherently prone to suicidality due to their sexual orientation or gender identity. Rather, there are general and LGBTQA+-specific risk factors, along with difficult or traumatic lived experiences that increase the likelihood of suicidality [34].

LGBTQA + students also began experiencing major depressive episode, and suicidal ideation at a significantly earlier age than their heterosexual counterparts. For instance, LGBTQA + students started to experience major depressive episodes at aged twelve, while heterosexual students began showing these symptoms at aged fourteen. Similarly, LGBTQA + higher education students also reported significantly earlier onset of suicidal ideation than their heterosexual peers, which is supported by existing international evidence [35]. These results demonstrate the importance of early identification and intervention, particularly for LGBTQA + young people who are experiencing these difficulties at a much earlier age. Critically, it has been contended that the mental health needs of LGBTQA + young people are not being met or adequately addressed, with support only arriving once a young person has already reached a crisis point [36]. These findings raise further concerns when taking into account the substantial evidence regarding reduced help-seeking behaviours among LGBTQA + young individuals [37, 38]. In addressing this, it should be noted that higher help-seeking intentions are strongly associated with more inclusive school environments, adequate supportive resources, and trusted adult support, which are all essential for improving help-seeking intentions and for early intervention or preventative care among LGBTQA + young people [39].

One of the most significant findings is arguably the discovery that LGBTQA + students encountered notably higher rates of childhood adversities and recent stressors than their heterosexual peers. More specifically, the findings identified that traumatic experiences of parental maltreatment such as physical abuse were up to three times higher among LGBTQA + higher education students. Furthermore, the prevalence of emotional abuse experiences among young people was nearly four times greater for LGBTQA + students (11.7%), than for heterosexual students (2.6%), which is particularly concerning. There were also significantly higher rates of parental maladjustment including parental mental illness, parental alcohol and/or drug misuse, and domestic violence. Notably, childhood adversities are widely recognised as significant predictors of non-suicidal self-injury and suicidal behaviour, with higher rates of childhood adversities reported within LGBTQA + populations [12, 40]. Another significant finding was that LGBTQA + higher education students felt less loved or cared for at home (45%), and less emotionally close to their family members (37%), than heterosexual students. This is clearly an impactful finding, perhaps best understood within the context of the elevated rates of childhood adversities which may have negatively impacted family relationships. However, it is important to also underscore that many LGBTQA + young people experience familial conflict, non-acceptance, and discrimination due to their sexual orientation and/or gender identity [41, 42]. Accordingly, government policies and educational programmes which focus on the prevention of childhood adversities, and the promotion of healthy relationships within the family may help reduce suicide risk among LGBTQA + higher education students.

For recent stressors, LGBTQA + students reported higher rates of interpersonal conflict, including within their friendships and family relationships. While the source of this relational conflict is unknown, family conflict and negative family treatment are significantly associated with suicidality among LGBTQA + youth [43]. LGBTQA + students also experienced twice as much bullying (20%), which is significant given that bullying, victimisation and peer rejection can have devastating consequences on a young person’s mental wellbeing [44]. It is unclear whether the bullying experienced by the LGBTQA + students was related to their sexual orientation or gender identity. However, in understanding LGBTQA+-specific risk factors for suicidality, the impact of queerphobia, societal stigma, and discrimination must be highlighted as important contributory factors to the disproportionate burden of mental health difficulties and suicidal distress [43]. Moreover, we must recognise that educational settings offer unique opportunities for the practice of inclusion and preventative care which can reduce the prevalence and impact of bullying among LGBTQA + students [45, 46]. In addition, it is concerning that LGBTQA + students reported experiencing higher rates of sexual harassment and sexual assault, compared to heterosexual students. Unfortunately, these findings are congruent with international literature on LGBTQA + sexual harassment and sexual assault, where a young person is targeted because of their sexual orientation and/or gender identity [47–49]. These findings clearly emphasise the importance of inclusive systems, policies, and formal processes within educational settings which must work to address sexual violence and provide specialised support for any higher education student who has experienced sexual harassment and abuse.

Interestingly, the analyses of multivariate logistic regression conducted on LGBTQA + students revealed that major depressive disorder, and probable post-traumatic stress disorder were significant predictors of suicidal thoughts and making a suicide plan, but not for suicide attempt. Although unexpected, it is clear that additional factors such as childhood adversities and recent stressors were more strongly associated with increased likelihood of suicide attempt, which is supported by existing literature [6, 50]. The finding that NSSI was significantly associated with increased likelihood of suicidal ideation, plan, and attempt among LGBTQA + students is well established by international evidence [11]. However, it is concerning that LGBTQA + students who reported NSSI were twelve times more likely to have made a suicide attempt in their lifetime, demonstrating the importance of effective interventions for NSSI [51]. Another important finding was that childhood adversities were significant predictors of suicide attempt among LGBTQA + higher education students. As discussed, a growing body of evidence has highlighted that LGBTQA + young people face heightened risk for childhood adversities [12, 40]. However, these findings exemplify the significance of early intervention and prevention, with trauma-focused therapies and supportive services for any young person who has experienced childhood adversities.

Overall health stress was also a significant predictor or risk factor for suicide ideation, plan and attempt, as well as NSSI among LGBTQA + students. As such, it should be acknowledged that LGBTQA + youth still face significant health inequalities in relation to both their physical and mental health [52]. Additionally, we know that a lack of LGBTQA + specific services within both educational settings and communities, including limited access to inclusive healthcare, are significant predictive factors for suicidality among LGBTQA + youth [53]. Certainly, limited access to inclusive healthcare represents an additional barrier for help-seeking among young people [54]. Significantly, the logistic regression analyses also identified that LGBTQA + students who had experienced bullying, at twice the prevalence rate of heterosexual students, were three times more likely to make a suicide attempt in their lifetime. Unfortunately, these novel findings are reflected within the lived experiences of students within educational institutions in Northern Ireland. For instance, it has been reported that 67% of all LGBTQ + student did not feel safe, welcomed, or valued at school, while 63% indicated that these experiences had negatively impacted their mental health [55]. In the United Kingdom, these adverse experiences extend into higher education, where just 37% of LGB students feel safe on university campuses, with additional challenges faced in rural regions [45, 56]. Consequently, there is an urgent necessity for effective measures and interventions to reduce incidences of bullying within educational settings, including discrimination and harassment policies which are inclusive of both sexual orientation and gender identity [57, 58].

### Limitations

Although the current research was conducted in a rigorous manner, a number of limitations should be considered when interpreting the overall results. First, only a subset of students studying in the ROI were recruited to the study and the sample may therefore not be representative of the whole student population. Despite this, all incoming first year students in NI were invited to participate. Additionally, moderately sized samples were obtained in both institutions and weights were applied to ensure that the analysis was representative in relation to both gender and age. Second, self-report surveys are always subject to certain biases and inherent limitations. For example, students may not have accurately reported all issues related to their mental health or their experiences, perhaps due to stigma or an unwillingness to disclosure such. Indeed, this may suggest that the prevalence rates could be even higher among all students, including LGBTQA + students. Third, probable PTSD was a screening item, based on self-reported symptoms and not a medical diagnosis. As such, it is possible that the prevalence rates for probable PTSD were higher than might be clinically relevant. Fourth, the original study was designed to focus on all higher education students, and not LGBTQA + students specifically. It follows then that there were specific areas of interest that could not be explored. For example, it would have been useful to explore if the increased rates of bullying were related to the student’s sexual orientation and/or gender identity. Fifth, for the multivariate analysis, two separate analyses were conducted, and consequently, it was not feasible to account for significant environmental variables when examining the associations between psychological distress variables and suicide-related outcomes. For future research endeavours, it could be useful to conduct a longitudinal study with a larger LGBTQA + sample in order to investigate significant within-group differences in terms of prevalence rates of mental health, childhood adversities, recent stressors, and suicidality among this population.

## Conclusions

Identifying, managing and treating mental health difficulties and suicidal behaviour among higher education students remains a challenge, and in the current study, there were considerable unmet mental health needs. Previous studies have highlighted that LGBTQA + higher education students were more likely to experience mental health disorders and suicidality than their heterosexual peers. However, it was not clear why this may be the case. To our knowledge this was the first study to examine mental health disorders, childhood adversities and recent stressors as risk factors for suicidality among LGBTQA + higher education students. In doing so, our findings emphasise the importance of early intervention and prevention of mental illness, the need for trauma-informed services due to higher rates of childhood adversities, and the necessity to tackle recent stressors such as bullying which confer significant risk for non-suicidal self-injury and suicidality. As discussed, educational settings offer unique opportunities for the practice of inclusion, preventive care and treatment which can help reduce the prevalence of mental health difficulties and suicidality among LGBTQA + higher education students.

## Data Availability

This study was conducted as part of the World Mental Health International College Student Initiative (WMH-ICS), and as per the relevant protocol, the data is not publicly available.
